# Supplementary material for: Multi-Phase, Contrast-Enhanced Computed Tomography-Based Radiomic Prognostic Marker of Non-Metastatic Pancreatic Ductal Adenocarcinoma
Source: Cancers (Basel). 2022 May 18;14(10):2476. doi: 10.3390/cancers14102476 (PMC9139570; doi:10.3390/cancers14102476)
Supplement: Supplementary file 1 [file cancers-14-02476-s001.zip › cancers-1696761-supplementary.pdf]

Article

Multi-Phase, Contrast-Enhanced Computed Tomography-Based Radiomic Prognostic Marker of Non-Metastatic Pancreatic Ductal Adenocarcinoma

Dong Woo Shin, Jaewon Park, Jong-Chan Lee, Jaihwan Kim, Young Hoon Kim and Jin-Hyeok Hwang

Table S1. Univariate and multivariate Cox hazard regression analysis of clinical and radiologic parameters in patients with borderline resectable or locally advanced pancreatic cancer on follow-up CT scan.

	Subgroup	Patients (%)	Median OS (95% CI) (months)	*p-value*	Univariate analysis		Multivariate analysis	
					HR (95% CI)	*p-value*	aHR (95% CI)	*p-value*
**Tumor size**	T1 (≤ 2 cm)	36 (25.9%)	20.5 (10.7–30.4)	<0.001	1 (reference)	-	1 (reference)	-
	T2 (2–4 cm)	63 (45.3%)	15.6 (11.7–19.5)		1.606 (0.944–2.732)	0.080	1.503 (0.880–2.566)	0.136
	T3 (>4cm)	40 (28.8%)	8.9 (7.8–10.1)		3.343 (1.904–5.871)	<0.001	2.825 (1.580–5.051)	<0.001
**UP**	<32.3 HU	35 (25.2%)	10.4 (6.7–14.1)	0.001	1 (reference)	0.001	1 (reference)	0.196
	≥32.3 HU	104 (74.8%)	16.1 (12.2–20.0)		0.474 (0.304–0.741)		0.628 (0.309–1.272)	
**PPP**	<48.6 HU	35 (25.2%)	8.9 (5.0–12.9)	0.007	1 (reference)	0.008	1 (reference)	0.009
	≥48.6 HU	104 (74.8%)	16.1 (12.9–19.3)		0.558 (0.363–0.859)		0.767 (0.479–0.952)	
**PVP**	<52.0 HU	22 (15.8%)	6.8 (4.0–9.7)	<0.001	1 (reference)	<0.001	1 (reference)	0.085
	≥52.0 HU	117 (84.2%)	16.1 (12.4–19.8)		0.383 (0.233–0.629)		0.550 (0.278–1.086)	

Abbreviations: BRPC, borderline resectable pancreatic cancer; LAPC, locally advanced pancreatic cancer; OS, overall survival; HR, hazard ratio; aHR, adjusted hazard ratio; CI, confidence interval; UP, unenhanced phase; PPP, pancreatic parenchymal phase; PVP, portal venous phase; HU: Hounsfield unit

Table S2. Correlation between tumor size and intra-tumoral contrast enhancement.

		RPC (n = 159)				BRPC/LAPC (n = 139)			
		**T1 (≤ 2 cm)**	**T2 (2–4 cm)**	**T3 (>4cm)**	***p-value***	**T1 (≤ 2 cm)**	**T2 (2–4 cm)**	**T3 (>4cm)**	***p-value***
**Initial CT**	UP (HU)	35.9 (28.5–43.3)	37.3 (35.8–38.8)	32.8 (30.5–35.2)	0.490	35.9 (28.5–43.3)	37.3 (35.8–38.8)	32.8 (30.5–35.2)	0.587
	PPP (HU)	72.3 (48.3–96.2)	65.9 (61.9–69.9)	48.9 (44.9–52.9)	0.056	72.3 (48.3–96.2)	65.9 (61.9–69.9)	48.9 (44.9–52.9)	0.052
	PVP (HU)	83.9 (52.8–114.9)	82.9 (77.5–88.3)	59.9 (54.7–65.2)	0.420	83.9 (52.8–114.9)	82.9 (77.5–88.3)	60.0 (54.7–65.2)	0.420
**Follow-up CT**	UP (HU)	NA	NA	NA	NA	40.5 (32.0–48.9)	37.8 (35.7–39.9)	35.2 (32.7–37.8)	0.851
	PPP (HU)	NA	NA	NA	NA	71.4 (54.2–88.6)	62.8 (58.9–66.7)	54.2 (49.0–59.4)	0.978
	PVP (HU)	NA	NA	NA	NA	94.6 (72.9–116.3)	82.1 (76.7–87.4)	66.3 (59.6–73.1)	0.972

Abbreviations: RPC, resectable pancreatic cancer; BRPC, borderline resectable pancreatic cancer; LAPC, locally advanced pancreatic cancer; CT, computed tomography; PPP, pancreatic parenchymal phase; HU, Hounsfield unit; NA, not applicable
